# Supplementary material for: Causal Relationship between Aging and Anorexia Nervosa: A White-Matter-Microstructure-Mediated Mendelian Randomization Analysis
Source: Biomedicines. 2024 Aug 16;12(8):1874. doi: 10.3390/biomedicines12081874 (PMC11351342; doi:10.3390/biomedicines12081874)
Supplement: Supplementary file 1 [file biomedicines-12-01874-s001.zip › Supplementary Figure/Figure S4/Singe SNP plot of MR analysis for TL to ubm-b-1590.pdf]

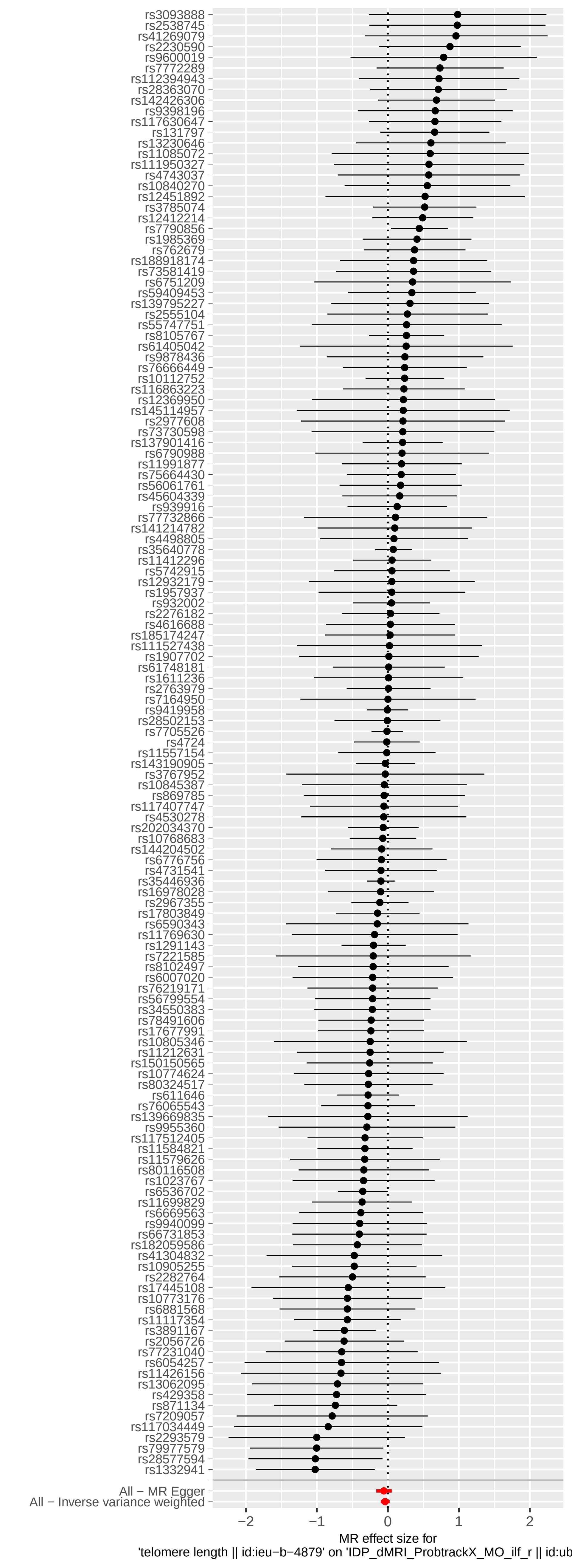
rs3093888
rs2538745
rs41269079
rs2230590
rs9600019
rs7772289
rs112394943
rs28363070
rs142426306
rs9398196
rs117630647
rs131797
rs13230646
rs11085072
rs111950327
rs4743037
rs10840270
rs12451892
rs3785074
rs12412214
rs7790856
rs1985369
rs762679
rs188918174
rs73581419
rs6751209
rs59409453
rs139795227
rs2555104
rs55747751
rs8105767
rs61405042
rs9878436
rs76666449
rs10112752
rs116863223
rs12369950
rs145114957
rs2977608
rs73730598
rs137901416
rs6790988
rs11991877
rs75664430
rs56061761
rs45604339
rs939916
rs77732866
rs141214782
rs4498805
rs35640778
rs11412296
rs5742915
rs12932179
rs1957937
rs932002
rs2276182
rs4616688
rs185174247
rs111527438
rs1907702
rs61748181
rs1611236
rs2763979
rs7164950
rs9419958
rs28502153
rs7705526
rs4724
rs11557154
rs143190905
rs3767952
rs10845387
rs869785
rs117407747
rs4530278
rs202034370
rs10768683
rs144204502
rs6776756
rs4731541
rs35446936
rs16978028
rs2967355
rs17803849
rs6590343
rs11769630
rs1291143
rs7221585
rs8102497
rs6007020
rs76219171
rs56799554
rs34550383
rs78491606
rs17677991
rs10805346
rs11212631
rs150150565
rs10774624
rs80324517
rs611646
rs76065543
rs139669835
rs9955360
rs117512405
rs11584821
rs11579626
rs80116508
rs1023767
rs6536702
rs11699829
rs6669563
rs9940099
rs66731853
rs182059586
rs41304832
rs10905255
rs2282764
rs17445108
rs10773176
rs6881568
rs11117354
rs3891167
rs2056726
rs77231040
rs6054257
rs11426156
rs13062095
rs429358
rs871134
rs7209057
rs117034449
rs2293579
rs79977579
rs28577594
rs1332941
All − MR Egger
All − Inverse variance weighted
−2
−1
0
1
2
MR effect size for
'telomere length || id:ieu−b−4879' on 'IDP_dMRI_ProbtrackX_MO_ilf_r || id:ub
